# Novel Organic-Inorganic Hybrid Polystyrene Nanoparticles with Trichromatic Luminescence for the Detection of Latent Fingerprints

**DOI:** 10.1155/2022/2230360

**Published:** 2022-03-07

**Authors:** Xiao Wang, Tao Liao, Haiyan Wang, Hongxia Hao, Qinglai Yang, Hong Zhou, Yu Ma, Minjie Zhi, Jiahao Wang, Ruihang Fan

**Affiliations:** ^1^Institute of Public Security, Northwest University of Political Science and Law, Xi'an 710122, Shaanxi, China; ^2^Shenzhen WWHS Biotech. Inc, Shenzhen 518100, China; ^3^Shanxi Datong University, College of Chemistry and Chemical Engineering, Datong, Shanxi 037009, China; ^4^Key Laboratory of Evidence Science, China University of Political Science and Law, Ministry of Education, Collaborative Innovation Center of Judicial Civilization, Beijing 100088,, China; ^5^Center for Molecular Imaging Probe, Cancer Research Institute, University of South China, Hengyang, Hunan 421001, China; ^6^Institute of Forensic Science, Ministry of Public Security, Beijing 100038, China; ^7^Nanjing Jinling Forensic Science Service, Nanjing 210019, Jiangsu, China

## Abstract

This article explored the application of novel organic-inorganic hybrid polystyrene nanoparticles (PSNPs) with trichromatic luminescence for the detection of latent fingerprints. The PSNPs were synthesized by encapsulated Eu(DBM)_3_phen, coumarin 6, and FDBT into the polystyrene nanoparticles through the swelling method and applied them to visualize latent fingerprints. The PSNPs had a spherical morphology with an average diameter of 310.7 nm, and they emitted trichromatic fluorescence (525 nm/570 nm/610 nm) under 365 nm excitation wavelength with green/yellow/red color under filters. They were less likely to aggregate, float or stain the background when treating fingerprints. The developed fingerprints with excellent clarity of ridges and contrast could be viewed, and the digital magnification of fluorescence-developed fingerprints provided more minutiae details about some regional patterns. The colorimetric and fluorescent trichromatic light could provide complementary signals without the background interference from fluorescent substrates and/or complex multicolor surfaces, which improved the applicability of fluorescent nanoparticles for fingerprints development. PSNPs are promising for the detection of latent fingerprints and practical criminal investigations with their ease of operation, eco-friendly properties, and excellent trichromatic optical performance.

## 1. Introduction

The corrugated skin at human fingers is characterized by a complex pattern of raised papillary ridges and depressed furrows. The papillary ridge patterns remain topologically unchanged from the birth of an individual and differ from one individual to another and differ from one finger to another [1]. Fingerprints represent the contact impression of the lifted papillary ridge of skin. Fingerprints analysis has been widely used as powerful and effective evidence for individual identification and recognition from ancient times since the patterns of the fingerprint ridge are unique for everyone and immutable throughout the whole life. It has provided reliable evidence for criminal investigations for more than a century due to the value of fingerprints in personal identification [2–5].

Fingerprints are an important class of evidence evaluated by law-enforcement agencies to assist with the identification of individuals [6]. But the most commonly found fingerprints are named latent fingerprints because such imprints are invisible to the naked eye. The exchange principle proposed by Edmond Locard states that every contact leaves a trace. Secreted eccrine sweat, oily sebum, lipids, and contaminants from the human body will leave an imprint of the finger's ridge patterns when a finger touches a solid surface, thus forming a latent fingerprint. Therefore, additional efforts are required to develop latent fingerprints for easy visualization.

A variety of efforts to develop rapid, sensitive, and economical techniques for the detection of latent fingerprints have emerged in the past few years [1]. To date, these techniques including powder dusting [7–11], fluorescent dye staining [12, 13], ninhydrin spraying [14], cyanoacrylate/iodine fuming [1, 15], vacuum metal deposition, and small particle reagent method [16–20] are the most widely used due to their simplicity, efficiency, and ease of operation. Although these traditional methods are effective under ordinary circumstances, there are still numerous challenges to visualize latent fingerprints with high contrast and low background interference on multicolor background, and there is an urgent need in seeking simple and efficient methods for developing the latent fingerprints with improved contrast, sensitivity, and selectivity. The fluorescence nanomaterials-based method is a general approach for latent fingerprints detection on the multicolor surface of object with high sensitivity [21–23]. Nanoparticle materials have attracted enormous interest with large surface area, enhanced stability, and high brightness [1, 24, 25]. The sensitivity of fluorescence detection usually is high even when the concentration of the analytes is very low. For example, quantum dots [26–28], metal colloids [29], and fluorescent upconversion nanoparticles [30–32] have been successfully utilized for the visualization of fingerprints [33–36]. However, typical fluorescent materials are usually monochromatic, which are difficult to be applied to the object with complex multicolor background and hard to distinguish the difference between fingerprint lines and multicolor background [34]. It is highly desirable to design a new and universal nanomaterial with high sensitivity, low background interference, and tunable luminescence color for fingerprint detection on objects with complex multicolor backgrounds [37].

Here, hybrid polystyrene nanoparticles (PSNPs) were prepared and characterized with trichromatic luminescence. We reported a novel strategy by using three different kinds of organic luminescent materials with different fluorescence emission and inorganic polystyrene nanoparticles to synthesize fluorescence nanomaterials with trichromatic luminescence, which could clearly visualize latent fingerprints on objects with complex multicolor background, such as plastic packaging bottles, ceramic tile, and flyer. We synthesized hybrid photoluminescent nanoparticles by encapsulated Eu(DBM)_3_phen (tris(dibenzoylmethane)-mono-(1,10-phenanthroline) europium(III)), coumarin 6, and FDBT (4,7-di-(7-(9,9-ditetradecyl-9H-fluoren-2-yl)-2,3-dihydrothieno[3,4-b][1,4]dioxin-5-yl)-2-(heptadecan-9-yl)-2H-benzo[d][1,2,3]triazole; the chemical structure was shown in the supporting materials) into the polystyrene nanoparticles through the swelling method and applied them to visualize latent fingerprints by a simple brushing method. All of the hybrid molecules have been proven to emit stable and bright fluorescence, possessing the potential for sensitive fingerprint visualization. After brushing the PSNPs powder onto the surfaces, the latent fingerprints revealed a canary yellow color and could be directly observed by the naked eyes under ambient light. The fluorescence of these PSNPs on the background was invisible to the naked eye under ambient light, and latent fingerprints could be fluorescently imaged under the excitation of a multiband light lamp with red/yellow/green luminous color with filters. Unlike conventional fingerprint visualization agents, PSNPs have several remarkable advantages for fingerprints detection, such as high fluorescent intensity, good chemical and photostability, good mechanical stability, facile synthesis, as well as tunable luminous color. The colorimetric and fluorescent trichromatic light could provide complementary signals simultaneously to minimize the background interference from fluorescent substrates and/or complex multicolor surfaces. The digital magnification of developed fingerprints provided clear minutiae details about some regional patterns without damaging the fingerprint details while brushing, such as the ridge starting, the bifurcation, the short ridge, the hook ridge, etc. PSNPs are promising for practical criminal investigations as a simple, fast, and accurate fingerprint development material.

## 2. Experimental Section

### 2.1. Chemicals and Materials

1,10-Phenanthroline (phen, 97%), europium chloride (III) hexahydrate (EuCl_3_·6H_2_O, 99.9%), methylene benzoyl (DBM, 98%), coumarin 6 (98%), acrylic acid (AA), dichloromethane, styrene, ethanol, potassium peroxydisulfate (KPS), and sodium hydroxide were purchased from Aladdin Reagent (Shanghai, China). Sodium dodecyl sulfate (SDS) and polyvinyl alcohol (PEG, Avg. 20000 Mw) were purchased from Sigma (St. Louis, MO, USA). FDBT was granted by Dr. Yang from the University of South China. All buffer solutions were prepared in the laboratory. The water was prepared via the Milli-Q (Millipore, Milford, MA, USA). The other chemical reagents were of analytical grade and obtained through standard commercial access.

### 2.2. Synthesis of Eu(DBM)_3_phen

Eu(DBM)_3_phen was synthesized following the previous reports [38]: 2 mmol phen (0.4 g), 6 mmol DBM (1.34 g), 6 mmol NaOH (0.24 g), and anhydrous ethanol (20 mL) were added to 100 ml round bottom flask under magnetic stirring, then heated at 50°C in a water bath. After 10 min, 2 mmol EuCl_3_·6H_2_O (0.52 g) dissolved in 20 mL ethanol was added drop wisely. Then 6 M NaOH was used to adjust the pH of the solution to 6-7. After stirring for 1 h, the precipitate was filtered off, washed with water and ethanol, and dried at room temperature to yield Eu(DBM)_3_phen.

### 2.3. Preparation of PSNPs

PSNPs were synthesized via encapsulating Eu(DBM)_3_phen, coumarin 6, and FDBT into monodisperse polystyrene nanoparticles solution. 50 mL suspension of carboxylic polystyrene nanoparticles (1%, w/v, dispersed in 0.25% SDS aqueous solution) was added to a 200 mL Erlenmeyer flask, then 10 mg Eu(DBM)_3_phen, 5 mg coumarin, and 10 mg FDBT dissolved in 5 mL CH_2_Cl_2_ was then added and sonicated for 10 min to form a uniform suspension. The resulted suspension was magnetically stirred at room temperature for 6 h, followed by heating at 50°C in a water bath for 48 h to completely evaporate the organic solvent CH_2_Cl_2_. The product was ultrasonically washed three times with ethanol and then three times with water by centrifugation. The resulting PSNPs were then dispersed in water (1% w/v) and kept at 4°C as stock. Then, take a few PSNPs solutions used for drying by the vacuum dryer and get PSNPs powder.

### 2.4. Fingerprint Collection and Development Process

To test the proposed materials, various nonporous-surfaced substrates (e.g., plastic packaging bottle, ceramic tile, and flyer) were examined. Before the fingerprints were deposited on the substrates, the donor should wash her/his hands in water and then rub the fingers from the oily parts of the body like the forehead region. After being carefully brushed with the prepared PSNPs powder, the latent fingerprints samples were irradiated with a multiband light lamp at 365 nm. The images of the fingerprints were observed and photographed through a digital camera (Nikon D7000, Japan) that was positioned above the sample surface with goggles.

### 2.5. Characterization

The surface profile characteristic of particles was studied using SEM (TESCAN MIRA3). The fluorescence spectrum was obtained by fluorescence spectrophotometer of Shimadzu Ltd. (RF-5301PC). The size of particles was characterized by the particle size characterization instrument of Malvern Instruments Ltd. (Zetasizer Nano ZSP). The excitation light source was a multiband light lamp (MCS-400Cɛ, Jobin Yvon, USA). The PSNPs powder was obtained by an electric blast drying oven (101-1A, Tianjin TAISITE, China).

## 3. Results and Discussion

### 3.1. Preparation of PSNPs

**Figure 1:**
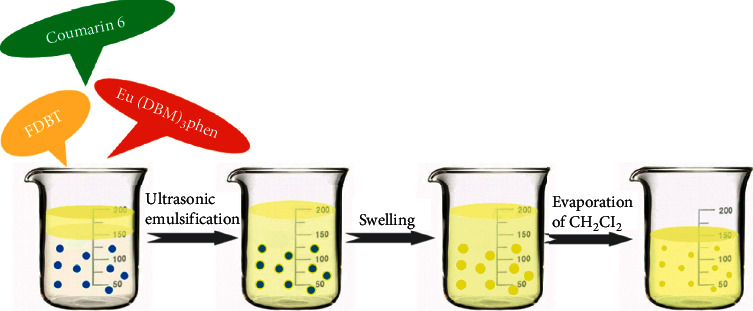
Schematic preparation of PSNPs through swelling method.

**Figure 2:**
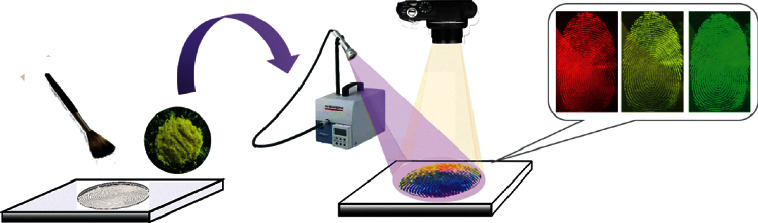
Visualization of latent fingerprints by brushing method.

Three major technical factors to be considered for the preparation of PSNPs: (1) water solubility or good dispensability of PSNPs in water; (2) formation of a stable complex with high thermodynamic stability and kinetic inertness; (3) high photochemical stability and quantum yield. We synthesized PSNPs by encapsulating Eu(DBM)_3_phen, coumarin 6, and FDBT into the polystyrene nanoparticles through the swelling method (Figure 1) and applied them to visualize latent fingerprints by a simple brushing method (Figure 2). Of the three fluorescent materials studied, Eu(DBM)_3_phen was synthesized according to our previous research procedures [38], coumarin 6 was obtained commercially and used as received, the FDBT was a newly synthesized fluorescent macromolecule with good fluorescence performance. The Eu(DBM)_3_phen was one of the lanthanide chelates that has been proven to emit stable and bright fluorescence, possess the lanthanide chelates' specific properties of a wide Stokes shift, sharp emission profiles, excellent stability against photobleaching, and long luminescence lifetime. Coumarin-6 was first employed as a fluorescent powder for developing the latent fingerprints along with the use of an argon ion laser in 1977. PSNPs were obtained through swelling prepared polystyrene nanoparticles with a mixture of Eu(DBM)_3_phen, coumarin 6, FDBT, and CH_2_Cl_2_ in microemulsion, followed by completely evaporating the organic solvent CH_2_Cl_2_, leaving Eu(DBM)_3_phen, coumarin 6, and FDBT precipitated within the hydrophobic polystyrene nanoparticles. The swelling method was easy to operate and effectively avoid the aggregation-induced fluorescence quenching, which was a universal problem for fluorescent nanomaterials when they changed from the liquid phase to the solid state. The carboxyl group modified on the polystyrene nanoparticles surface could be easily modified with other functional groups to form a stable bond for more uses, such as the specific identification of fingerprints.

**Figure 3:**
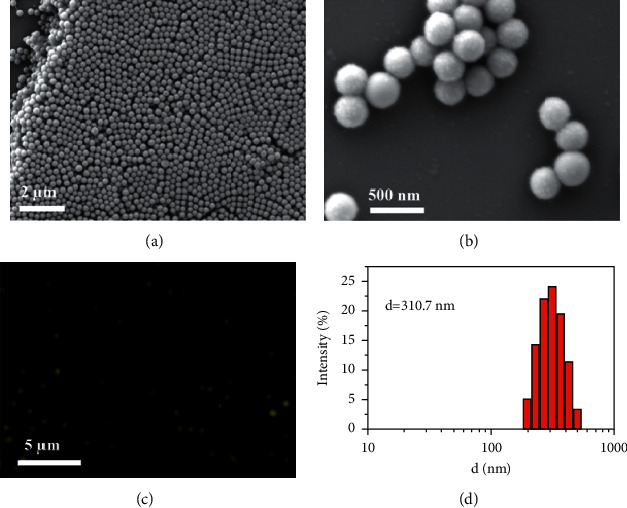
Characterization results of the PSNPs. The SEM images of polystyrene nanoparticles before (a) and after encapsulation of Eu(DBM)_3_phen, coumarin 6, and FDBT (b). Fluorescence microscope image in aqueous (c). The dynamic light scattering (DLS) spectrum, the nanoparticles mean diameter is 310.7 nm (d).

**Figure 4:**
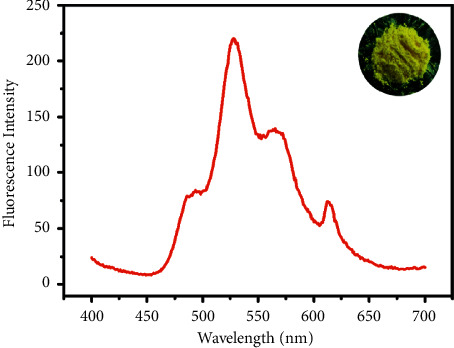
The fluorescence emission spectra of the PSNPs. The inset in Figure 4 shows a photograph of PSNPs under ambient light.

Scanning electron microscope (SEM) was used to investigate the structure and morphology of the PSNPs, as shown in Figures 3(a) and 3(b). From the SEM image of the synthesized PSNPs, we can observe that PSNPs form monodisperse quasispherical nanoparticles with an average size of about 310 nm. The size distributions were further confirmed by dynamic light scattering (DLS) analysis (Figure 3(d)). According to these results, the PSNPs have a narrow distribution with an average diameter of 310.7 nm. Also, PSNPs were stable in water and no apparent aggregation or sediment was observed at room temperature. In addition, PSNPs were highly monodisperse, and no apparent changes of surface topography could be observed after encapsulation of Eu(DBM)_3_phen, coumarin 6, and FDBT. The fluorescence microscope image taken at higher magnification in aqueous was shown in Figure 3(c) demonstrating that fluorescence can be excited from them.  Figure 4 depicts the fluorescence spectrum of PSNPs. The inset in Figure 4 shows a photograph of PSNPs powder under ambient light that revealed a canary yellow color. The dilute aqueous solution of the PSNPs exhibited three kinds of emission spectra under an excitation of 365 nm. The PSNPs emitted fluorescence emission around 525 nm, which was according to coumarin 6, 570 nm, which was according to FDBT and 610 nm, which was according to Eu(DBM)_3_phen under UV excitation. It was supposed that the red shift of optimal emission wavelengths should be owing to the growth of molecular rigidity of PSNPs.

### 3.2. Application for Fingerprints Detection

The PSNPs fluorescent powder can be extensively employed for developing fingerprints on various multicolor substrates. Nonporous-surface substrates with bright multicolor were chosen, such as plastic packaging bottles, ceramic tile, and flyers. Before the fingerprints were deposited on the substrates, the donors should wash her/his hands in water and then rub the fingers from the oily parts of the body and further visualize the latent fingerprints by powder brushing technique. The powder brushing method is one of the oldest and more prevalently applied methods for latent fingerprint development on nonporous substrates. It has been in use since the late 19th century. It is efficient and cost-effective and could yield apparent fingerprints instantly on almost all smooth and nonporous substrates. In the powder brushing method, the fingerprint powder particles mechanically or physically adhere to the aqueous or oily components present in the latent fingerprint residues [1]. The powder brushing method involves a few simple steps: (1) latent fingerprint was deposited on a substrate; (2) PSNPs powder was sprinkled on the fingerprints, and the powder was spread over the full area of the marks using a smooth brush; (3) excess powder was removed initially with the air flow.

**Figure 5:**
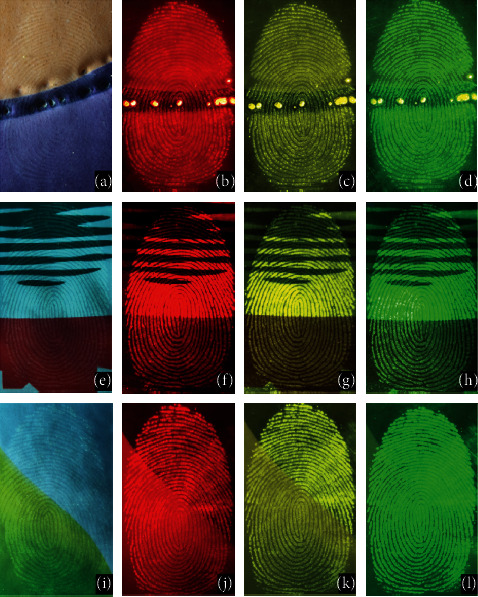
Digital photos of a fluorescence-developed fingerprints on ceramic tiles (a–d); Plastic packaging bottle(e–h); flyer (i–l). Figures (a), (e), and (i) were taken under ambient light and the others were taken under multiband light (365 nm) with filters.

It can be seen from Figure 5 that the PSNPs revealed high quality images which showed well-resolved ridge patterns that meet the requirements for individual identification for forensic purposes. Developed fingerprints were visualized under the multiband light lamp and well-defined, clear images of fingerprints were easily captured in this case under 365 nm excitation. The fingerprints deposited on these multicolor surfaces showed a high contrast under bright field illumination and could be clearly detected with different colors of lines, similar to the fingerprint development results on the single background color materials. Our PSNPs fluorescent powder could be used for developing the latent fingerprints with low background interference. The digital images were captured in a dark room and in the presence of ambient light. In both the conditions, fingerprints were of high contrast and clearly visible, the lines and furrows were clearly distinguished. Figures 5(a)–5(d) were taken on the multicolor ceramic tile; figures 5(e)–5(h) were taken on the plastic packaging bottle; figures 5(i)–5(l) were taken on the flyer. Figures 5(a), 5(e), and 5(i) were the digital pictures photographed taken under ambient light, and the others were taken under multiband light with filters. Multiband light lamp was used for the visualization of fingerprints in red/yellow/green color when excited by 365 nm UV irradiation. The contrast was enhanced markedly by the red/yellow/green fluorescence and the red fingerprints (Figures 5(b), 5(f), and 5(j)) were material Eu(DBM)_3_phen itself, whose wavelength 610 nm was the corresponding characteristic emission peak; the yellow fingerprints (Figures 5(c), 5(g), and 5(k)) were material FDBT itself, whose wavelength 570 nm was the corresponding characteristic emission peak; the green fingerprints (Figures 5(d), 5(h), and 5(l)) were material coumarin 6 itself, whose wavelength 525 nm was the corresponding characteristic emission peak. The PSNPs with tunable trichromatic light were very beneficial for the fingerprint displaying and distinguishing on complex multicolor surfaces. Due to the small size, the surface-to-volume ratio was very high, which improved the attachment of particles with the latent fingerprints, even very weak fingerprints.

### 3.3. Digital Magnifications of Fluorescence-Developed Fingerprints

**Figure 6:**
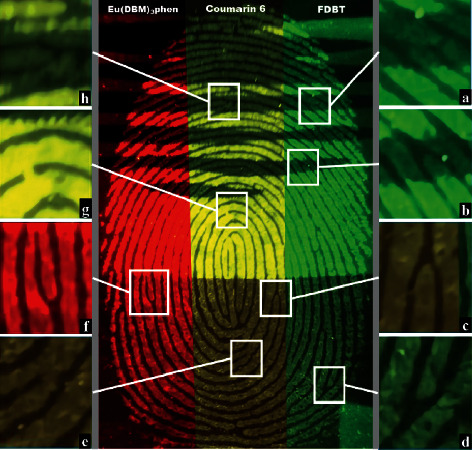
Digital photos of a fluorescence-developed fingerprints and magnified regional images with some specific minutiae details. (a, e) Bifurcations; (b, c) starting ridges; (d, h) ending ridges; (g) short ridge; (f) hook ridge.

The minute details analysis of latent fingerprints is essentially required in forensic investigations, which presents the invisible problems in any crime scene. The high quality of the fingerprint images obtained using this technique enables visualization of primary level minutiae and second level minutiae such as bifurcation, crossover, and termination structures, etc. The digital magnifications of developed fingerprints in Figure 6 were good enough to mark the nature of curves in fingerprint (series of ridges and furrows on the surface of the finger). The fingerprints contained many minute details like bifurcations, islands, enclosures, which are called Galton details. The number and position of these minute details vary from person to person and are very important for individualization. The minutiae details found in the latent fingerprint image were marked with white circles in Figure 6 from (a) to (h). The ridge starts are shown in Figures 6(b) and (c); the ridge endings are shown in Figures 6(d) and (h); the bifurcations are shown in Figures 6(a) and (e); the short ridge is shown in Figure 6(g), and the hook ridge is shown in Figure 6(f). The high-magnification optical images from Figure 6 revealed that the powder was attached only to the ridges, and the grooves were empty and did not show any emission color. The successful identification of the detailed substructures indicated the high sensitivity and reliability of the PSNPs for rapid fingerprint development. The obtained results indicated that the prepared solid state luminescent PSNPs were useful as fingerprint labeling agents due to their strong photo-luminescence and photostable properties.

## 4. Conclusions

Although a couple of luminescence materials have been reported in the literature recently about fingerprints detection, the challenges remain to increase the sensitivity and luminescence versatility of these materials for the application. This paper reported the realization of the development of the latent fingerprint with novel organic-inorganic hybrid polystyrene nanoparticles with trichromatic luminescence, which was one-step synthesized using Eu(DBM)_3_Phen, coumarin 6, FDBT, and polystyrene nanoparticles. The experiments showed that Eu(DBM)_3_Phen, coumarin 6, and FDBT individually show excellent emission, respectively, and the acquired fluorescence fingerprint images provide clear and coherent ridges, enough minutiae signatures (series of ridges and furrows on the surface of the finger), and strong fluorescence intensity. Under 365 nm excitation, emission peaks 525 nm, 570 nm, and 610 nm were observed in the fluorescence spectrum, which indicates an effective coupling between Eu(DBM)_3_Phen, coumarin 6, FDBT, and polystyrene nanoparticles complex in PSNPs. It has demonstrated that PSNPs could effectively develop the latent fingerprints on a variety of substrates with multicolor surfaces and solve the problem of recognizing developed fingerprints on complex multicolor surface because of the tunable trichromatic luminescence. PSNPs have been proven to hold great promise for latent fingerprint detections on complex multicolor surfaces and practical criminal investigations with their ease of operation, eco-friendly properties, and excellent tunable optical performance, and help to narrow the range of suspects during criminal investigations and forensic science.

## Data Availability

The data including scanning electron microscope images, fluorescence microscope images, dynamic light scattering spectrum, fluorescence emission spectra, and digital photos data used to support the findings of this study are included within the article and the supplementary information file. The data used to support the findings of this study have not been made available because there are no data about chromatography or mass spectrometry analysis.

## Supplementary Materials

Supplementary Materials The supplementary description is the chemical structure of FDBT: 4,7-di-(7-(9,9-ditetradecyl-9H-fluoren-2-yl)-2,3-dihydrothieno[3,4-b][1,4]dioxin-5-yl)-2-(heptadecan-9-yl)-2H-benzo[d][1,2,3]triazole.
