# Supplementary material for: Novel Organic-Inorganic Hybrid Polystyrene Nanoparticles with Trichromatic Luminescence for the Detection of Latent Fingerprints
Source: Int J Anal Chem. 2022 Mar 7;2022:2230360. doi: 10.1155/2022/2230360 (PMC8920635; doi:10.1155/2022/2230360)
Supplement: Supplementary Materials — The supplementary description is the chemical structure of FDBT: 4,7-di-(7-(9,9-ditetradecyl-9H-fluoren-2-yl)-2,3-dihydrothieno[3,4-b][1,4]dioxin-5-yl)-2-(heptadecan-9-yl)-2H-benzo[d][1,2,3]triazole. [file 2230360.f1.pdf]

Supporting materials

The chemical structure of FDBT:

4,7-di-(7-(9,9-ditetradecyl-9H-fluoren-2-yl)-2,3-dihydrothieno[3,4-b][1,4]dioxin-5-yl)-2-(heptadecan-9-yl)-2H-benzo[d][1,2,3]triazole

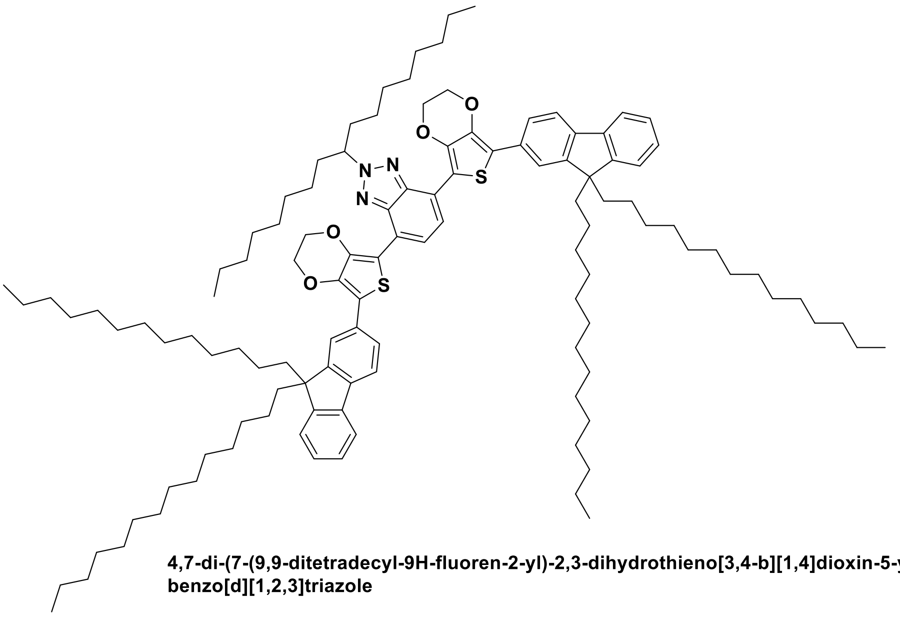

4,7-di-(7-(9,9-ditetradecyl-9H-fluoren-2-yl)-2,3-dihydrothieno[3,4-b][1,4]dioxin-5-yl)-2-(heptadecan-9-yl)-2H-benzo[d][1,2,3]triazole

Molecular Weight: 1751.82
